# Diagnostic accuracy of point-of-care devices for detection of anemia in community settings in India

**DOI:** 10.1186/s12913-020-05329-9

**Published:** 2020-05-26

**Authors:** Sutapa Bandyopadhyay Neogi, Jyoti Sharma, Shivam Pandey, Nausheen Zaidi, Maitreyee Bhattacharya, Rakhee Kar, Sitanshu Sekhar Kar, Abhishek Purohit, Sanjib Bandyopadhyay, Renu Saxena

**Affiliations:** 1 Indian Institute of Public Health- Delhi, Delhi NCR, India; 2 Calcutta Medical College, Kolkata, India; 3 JIPMER, Puducherry, India; 4 AIIMS, Jodhpur, India; 5 AIIMS, New Delhi, India

**Keywords:** Anemia, Hemoglobin, Diagnosis, Community, Invasive, Non invasive, Devices

## Abstract

**Background:**

Accurate diagnosis of anemia by community workers using a point-of-care device is a challenge. The objective of the study was to establish the diagnostic accuracy of point-of-care devices for detecting anemia in community settings.

**Methods:**

It was diagnostic accuracy study with cross-sectional design on adult patients attending the outpatient department of rural/ urban health centres of Medical colleges from India. The index tests were HemoCue, TrueHb, Massimo’s device and spectroscopic device, compared against autoanalyzer (gold standard). Accuracy was expressed by sensitivity, specificity, likelihood ratios, predictive values, area under the curve (AUC) and levels of agreement. For the diagnostic accuracy component, 1407 participants were recruited with a minimum of 600 for each device. An additional 200 participants were considered to elucidate the performance of devices in different weather conditions.

**Results:**

HemoCue and TrueHb performed better than Massimo and spectroscopic devices. Detection of anemia by technicians was similar between TrueHb and HemoCue (AUC 0.92 v/s 0.90, *p* > 0.05). Community workers performed better with Hemocue for detecting anemia compared to TrueHb (AUC 0.92 v/s 0.90, *p* < 0.05). For detection of severe anemia, accuracy of TrueHb was significantly better with technicians (AUC 0.91 v/s 0.70; *p* < 0.05) and community workers (AUC 0.91 v/s 0.73; *p* < 0.05).

HemoCue showed a bias or mean difference (95%CI) of 0.47 g/dl (0.42, 0.52) for all values, and 0.92 g/dl (0.82, 1.03) for severe anemia. For TrueHb, it was − 0.28 g/dl (− 0.37, − 0.20) for all readings, and 0.06 g/dl (− 0.52, 0.63) for severe anemia. TrueHb appeared to be more consistent across different weather conditions, although it overestimated Hb in extreme cold weather conditions.

**Conclusion:**

For detection of anemia, True Hb and HemoCue were comparable. For severe anemia, True Hb seemed to be a better and feasible point-of-care device for detecting anemia in the community settings.

## Key messages

- A valid, easy to use point-of-care device is required for detection of anemia in the community
- Several devices are available that requires validation through large scale community based studies
- Of all the devices tested, HemoCue and TrueHb devices fared better than Massimo and spectroscopic devices
- For detection of anemia, both HemoCue and TrueHb were comparable while for severe anemia, TrueHb performed better than HemoCue
- Both the devices overestimated Hb by 2 g% in extreme cold weather conditions

## Background

Anemia remains a public health challenge even today in India [1]. Hemoglobin (Hb) estimation is the cornerstone for diagnosis of anemia. It is the basis for instituting preventive and therapeutic interventions and measuring the outcome of management. Several methods are available for estimation of Hb [2–5]. However, the choice of methods depends on site of use (clinical/ community settings), availability of resources, validity of the device, the user (technicians/ health workers/ nurses), and population (adults/ blood donors/ children/ pregnant women), to name a few.

Appropriate treatment relies on accurate diagnosis at the point of care. Different devices for detection of anemia have been evaluated in the country [4, 6–9]. A device for use in communities or primary healthcare facilities in resource-poor settings should be inexpensive, rapid, and easy to perform with reasonable accuracy. Operational issues also aid in taking a decision on the feasibility of its use in field settings. Some devices that have the potential for use in field settings in India include invasive devices (require finger pricking for taking blood - HemoCue, TrueHb) and non- invasive devices (do not require pricking -Massimo’s device and AJO spectroscopic device). HemoCue and Massimo’s device have been validated and extensively evaluated in field settings [6, 10–16]. The findings are however not very consistent [12, 15–17].

There is a need to have a point of care diagnostic device that can detect anemia with reasonable accuracy, especially in lower and middle income countries, where the burden of anemia is high. For the national Anemia Mukt Bharat (Anemia Free India) programme, diagnosis of anemia remains the mainstay for further management [18]. Its diagnosis in primary care public health settings is usually done by community workers (female health workers or Auxillary Nurse Midwife or ANM). We therefore, aimed to establish the diagnostic accuracy of the devices for measurement of Hb and identify the most valid device for inclusion in the program.

The study was conducted with the objective of establishing the diagnostic accuracy of point-of-care devices (-HemoCue, TrueHb, Massimo’s device, and spectroscopic device) against automated analyzers (gold standard) for detecting anemia in community settings.

The secondary objectives were to establish the level of agreement in the classification of anemia as reported by ANM (using the devices that were found better) and laboratory technician and to assess the performance of devices in different weather conditions.

## Methods

### Study design

The study was done in two phases. The first phase was a diagnostic accuracy study with cross sectional design conducted at field practice areas of tertiary care hospitals from India. It aimed at determining the performance (diagnostic accuracy) of every device and level of agreement between technicians and frontline workers. The second phase, also a cross sectional study assessed the accuracy of devices in different weather conditions.

### Study settings

In India, field practice sites from rural and urban areas are attached to every Medical college. For the first phase of the study, we included rural/ urban field practice areas of two Medical colleges (Jawaharlal Institute of Postgraduate Medical Education and Research (JIPMER), Puducherry and Calcutta Medical College, Kolkata, West Bengal). For the second phase of the study, All India Institute of Medical Sciences (AIIMS) Jodhpur and Rural hospital, Reckong Peo (Himachal Pradesh) were considered. The sites were considered depending on the willingness of the Head of the Institution/ Department of Community Medicine to participate in the study and with facilities for performing the reference test.

The data collection for the study was conducted between August 2018 and March 2019.

#### Study participants

The study population included all adult patients attending the outpatient departments of urban or rural health centres of study sites for routine investigations. Consecutive patients who underwent any haematological investigation as advised by the on-duty medical officer (who was independent of the study) were considered. Patients (more than 18 years and less than 60 years) who gave informed consent were recruited in the study. Pregnant women, children, seriously ill patients and those with known bleeding diathesis were excluded due to ethical reasons.

### Test methods

Hematological auto-analyzer was selected as the reference test (***gold standard***) [4]. This system is an automated blood cell counter which measures Hb using non-cyanide method. The automated cell counters certified by external quality check were used as gold standard for the study.

Index tests included HemoCue 301, TrueHb version 1.2, Massimo non - invasive pulse oximetry version and AJO Spectroscopic device, version 1.0. Standard protocols were followed to conduct the testing. For Invasive tests, Hb concentration was measured using capillary and venous blood samples. The details of the devices are described elsewhere [19].

#### Main outcomes

The diagnostic accuracy of the devices were expressed in terms of sensitivity, specificity, predictive values, likelihood ratios and Area under the curve (AUC).

#### Sample size

nMaster 2.0 software was used to estimate sample size. Sample for the first phase (diagnosis accuracy study) was calculated using prevalence of anemia as 50% [4], sensitivity of  82% [4, 5], and 5% level of significance. A sample size of 600 was considered adequate to assess the diagnostic accuracy of every device [19].

To assess the performance of the devices in different weather conditions (second phase of the study), we assumed a population agreement of 0.7, sample agreement of 0.9, prevalence of 30% obtained from first phase of the study, power 90%, for two sided test at 5% level of significance. The required sample size was 156 that was rounded off to 200.

### Data collection and management

For the purpose of the study, each patient underwent two index tests (one invasive and the other non invasive) apart from the reference test. A schedule was prepared with alternate days earmarked for the devices. On any given day, the same set of devices were used for all patients to avoid any bias. The index tests were always performed before the reference test. The blood samples for the index tests and the reference tests were collected in the same sitting and there was no treatment administered in between. For the venous sample and autoanalyzer readings, the same sample was used.

Different methods were followed for collection of data for the index and reference tests. Data collection for index tests was done by three research staffs (qualified medical laboratory technicians) and four frontline workers per site. They were trained on the study protocol. They recruited the eligible patients after taking written informed consent, performed the index tests (one invasive using venous blood and one non-invasive), entered data in a tablet directly and transferred data to the investigators daily. The form was inbuilt in a tablet uploaded with an android based mobile application tool Census and Survey Processing System (CSPro). Checks were in-built within the system to track any tampering with the data, if at all it was there.

Four ANMs or frontline workers performed the index tests in a separate room (one invasive using capillary sample and one non-invasive) on the same patient in the same sitting. Data were entered in paper forms by frontline workers and sent to investigators every week.

To ensure blinding, the reference test (by using autoanalyzer in this case) was performed by the laboratory technician of the haematology laboratory who was independent of the study team. Data on reference test were collected by Site Investigators on paper forms and transferred electronically to the Investigators. Data from both the sources was then matched based on the unique Identification number given to each study participant and merged with the main data.

### Data analysis

Data were exported to STATA SE 11 and SPSS version 19.0 for analysis. A descriptive analysis was done for the key variables and continuous variables presented as means and categorical variables as proportions. Each of the index tests (categorized by venous and capillary blood for invasive tests) was compared against the Gold standard.

The study population was classified into no anemia (Hb >/= 11.0 g/dl), mild (Hb 10.0–10.9 g/dl), moderate (Hb 7.0–9.9 g/dl) and severe anemia (Hb < 7.0 g/dl) based on Indian Council of Medical Research (ICMR) classification. The first set of analysis focussed on screening of anemia. For this, mild, moderate and severe anemia was combined into a single category and was compared with no anemia. In the second set of analysis, no and mild anemia were clubbed as a single category and compared against moderate and severe anemia combined. The rationale for this classification was to identify patients who need treatment for anemia. In the third set of analysis, severe anemia was compared against mild, moderate and no anemia taken together. Identification of severe anemia is a priority that requires immediate attention and follows a different management protocol and hence this categorization.

Missing data or in determinant data of the index test or reference standards were excluded from the analysis.

The diagnostic accuracy of the devices were expressed in terms of sensitivity, specificity, predictive values, likelihood ratios and AUC. All the devices were compared with each other for sensitivity, specificity and AUC for technicians and ANM separately using chi-square test. Bland Altman test was done to assess the level of agreement between reference test and index tests. Differences in means of Hb level of patients between index tests and gold standard (risk of bias and limits of agreement) as measured on a continuous scale were reported.

The levels of agreement between the technicians (venous) and ANMs (capillary) were measured for the devices that were found to be accurate (sensitivity > 75% and specificity > 75%) and reported as kappa statistics.

The reproducibility was assessed by comparing the results of repeated examinations of the same patient. The level of agreement between research staff and the site coordinator with similar background (both technicians) was measured on a subsample of patients (100) for every device. For invasive devices, venous blood was used. The level of agreement was anlayzed by doing the Bland Altman test.

Reporting of results are based on STARD guidelines.

### Quality assurance

All study staffs were trained for data collection process and device operation in a common workshop. The facilitators included investigators as well as the technical experts from companies who supplied the devices. The device manufacturers certified the participants on their successful completion of training. On site monitoring and trainings were conducted by the facilitators. A single autoanalyzer in each site was earmarked for the reference test. Internal Quality Checks of the autoanalyzers were done every day. External Quality Assurance (EQAS) mechanisms, which is coordinated by AIIMS, New Delhi, were in place in all the sites. The samples are sent by AIIMS routinely to the sites every month. These are simultaneously assessed at AIIMS and concordance reports generated to ensure quality.

### Ethical considerations

Written informed consent was obtained from every eligible patient. Information collected did not have access to anyone else other than the research team. The treatment of patients was based on the readings of the reference test and was in no way influenced by the results of the index test. Approvals were obtained from All India Institute of Medical sciences (AIIMS), New Delhi [IEC/NP-416/09.10.2015, OP-2/09.02.2017, OP-19/29.12.2017), Indian Institute of Public Health, Delhi (IIPHD-IEC-03/2014 dated 21/04/2018),Medical College, Kolkata (MC/KOL/IEC/NON-SPON/65/04–2018), JIPMER, Puducherry (JIP/IEC/2018/0288) .

#### Patient and public involvement

This research was done without patient involvement. Patients were not invited to comment on the study design and were not consulted to develop patient relevant outcomes or interpret the results. Patients were not invited to contribute to the writing or editing of this document for readability or accuracy.

## Results

### Results of diagnostic accuracy (first phase)

**Fig. 1:**
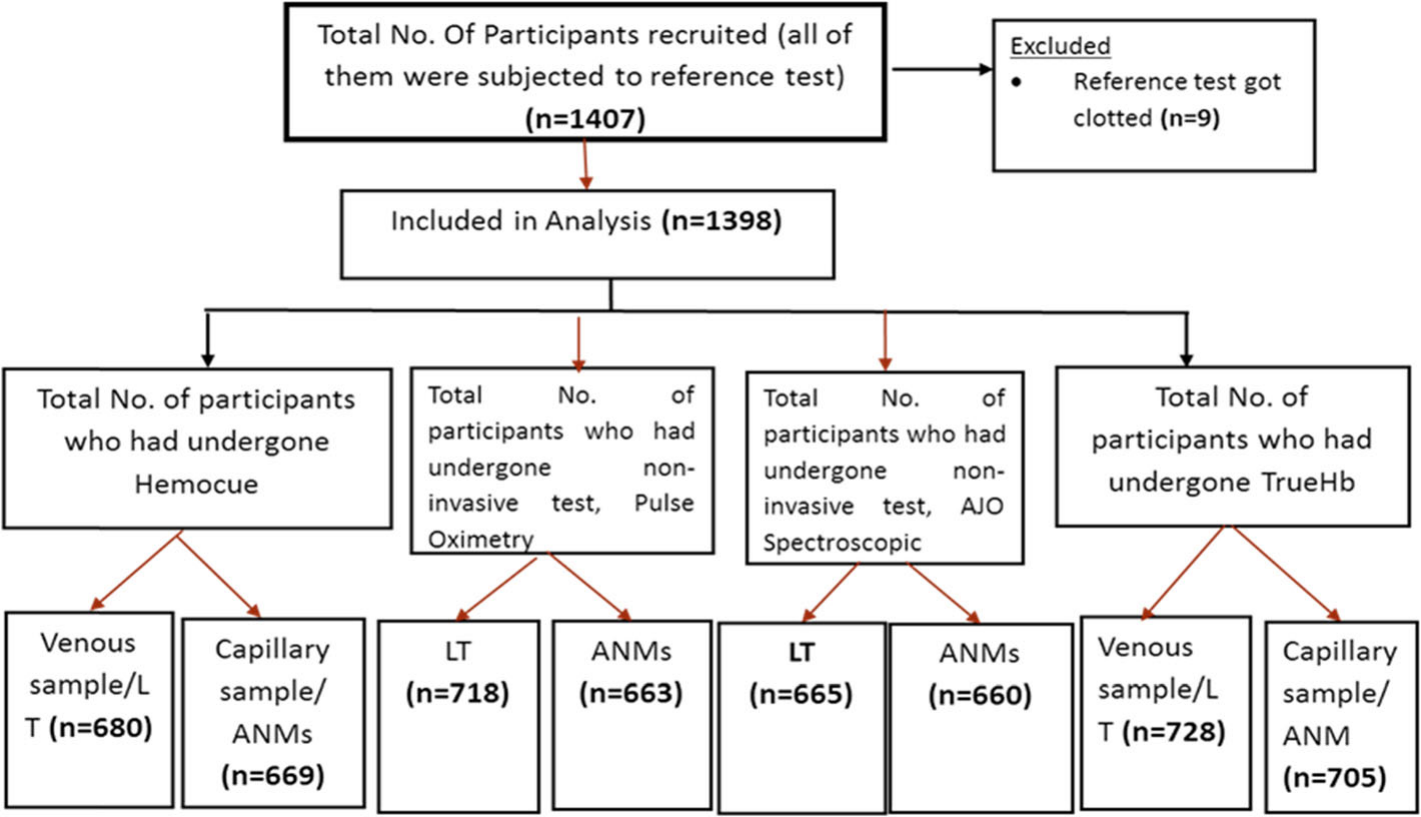
Flowchart depicting recruitment of participants in the study for various tests

A total of 1407 participants were recruited from the two sites over a period of 6 months (Fig. 1). Nine (< 1%) participants’ readings were missing (owing to clotting of blood) and hence excluded. There were no adverse events reported from the conduction of these tests.

The site wise distribution of patients was equitable. The mean age of the participants was 37.26 years (SD- ± 12.57 years). Out of 1407 participants whose presenting complaints were recorded, hematological disorder (13.9%), malignant conditions (12.5%), endocrinal (14.5%) and cardiovascular (9.5%) were the commonest provisional diagnosis. As per the ICMR classification, prevalence of anemia (Hb < 11 g/dl) in the study population was 33%. The distribution of anemia among the participants is included in Table 1.

**Table 1:** Distribution of study participants as per ICMR classification of anemia across two sites

Hb (gm/dl)	Total (***n*** = 1398)	Puducherry (***n*** = 752)	Kolkata (***n*** = 646)
**Mean (SD)**	11.64 (±2.7)	12.31 (±2.4)	10.8 (±2.8)
**Range**	2–20.2	2–20.2	4.2–18.6
**No anemia (Hb>/=11)**	938 (67.1%)	580 (77.1%)	358 (55.4%)
**Mild anaemia (Hb 10–10.9)**	124 (8.8%)	58 (7.7%)	66 (10.2%)
**Moderate anaemia (Hb 7–9.9)**	240 (17.2%)	94 (12.5%)	146 (22.6%)
**Severe anaemia (Hb < 7)**	96 (6.8%)	20 (2.6%)	76 (11.7%)

**Fig. 2:**
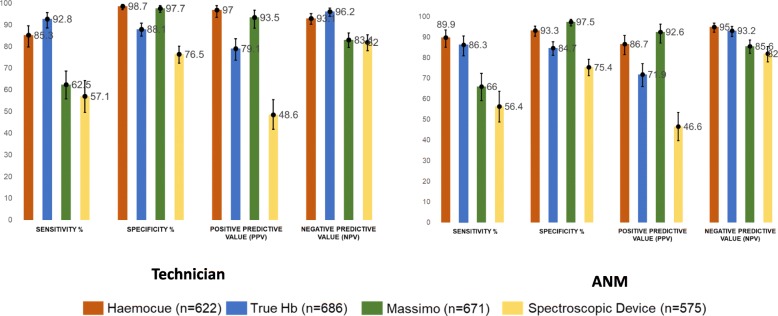
Diagnostic Accuracy Parameters for testing anemia and no anemia as per ICMR classification

**Fig. 3:**
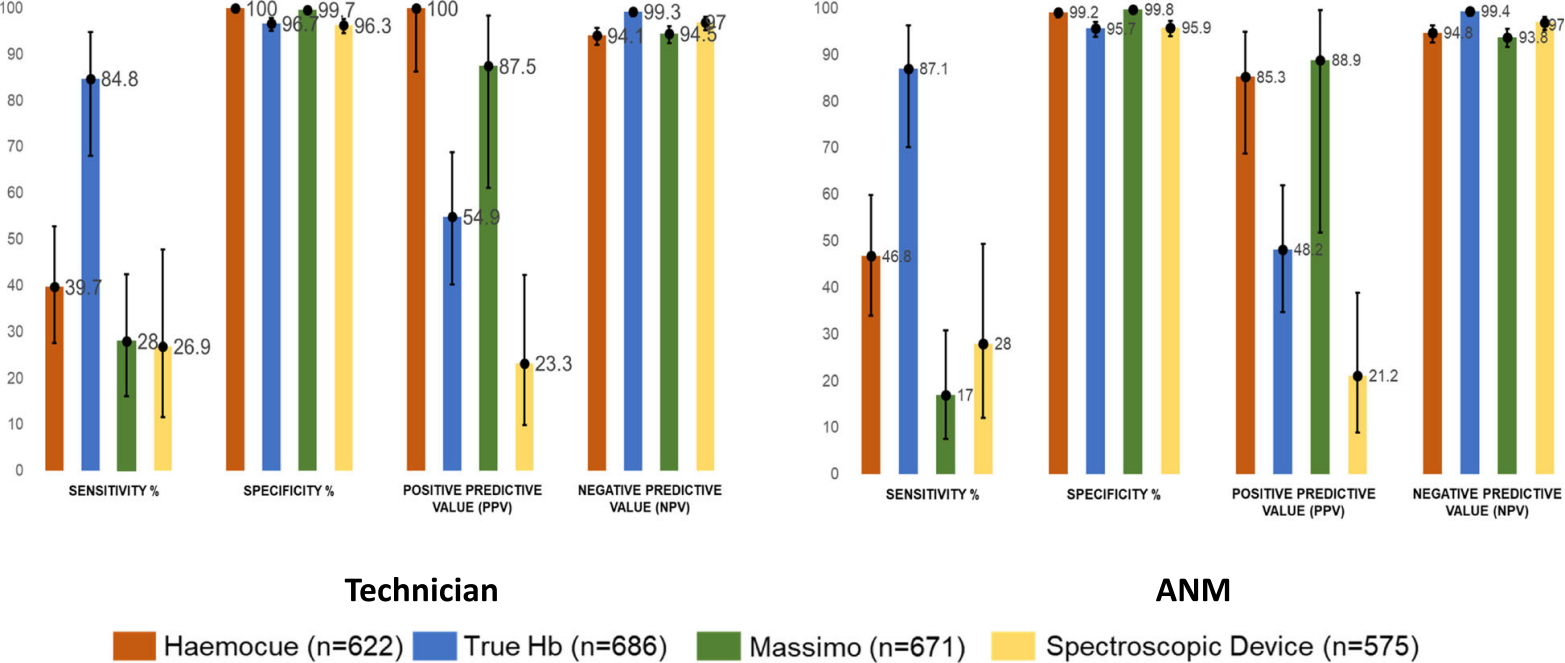
Diagnostic Accuracy Parameters for testing severe anemia as per ICMR classification

For technicians, the accuracy of HemoCue and TrueHb was found to be better compared to Massimo and spectroscopic devices. Sensitivity was significantly higher for TrueHb (92.8% v/s 85.3%) while specificity was higher for HemoCue (98.7% v/s 88.1%). Overall, detection of anemia by technicians was similar between TrueHb and HemoCue (AUC 0.92 v/s 0.90, *p* > 0.05). Community workers performed better with Hemocue compared to TrueHb (AUC 0.92 v/s 0.90, *p* < 0.05). However, for detection of severe anemia, accuracy of TrueHb was significantly better than HemoCue with technicians (AUC 0.91 v/s 0.70; *p* < 0.05) as well as community workers (AUC 0.91 v/s 0.73; *p* < 0.05). The non-invasive devices showed reduced sensitivity when tested for severe anemia. (Figs. 2, 3; Additional file 1: Webtable 1, 2).

**Fig. 4:**
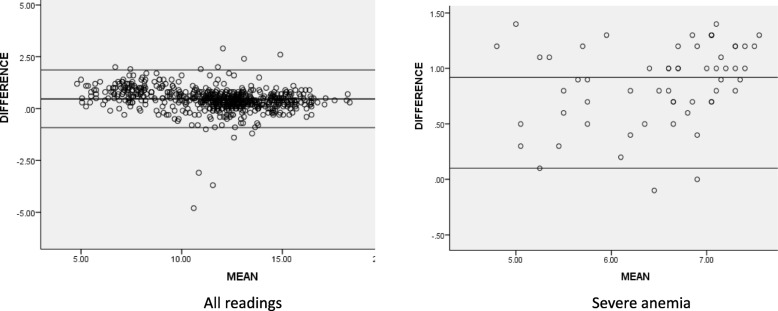
Bland-Altman Plot (Concordance analysis) showing agreement between auto-analyser V/s venous sample for Hemocue (Hb values expressed as gm/dl)

**Fig. 5:**
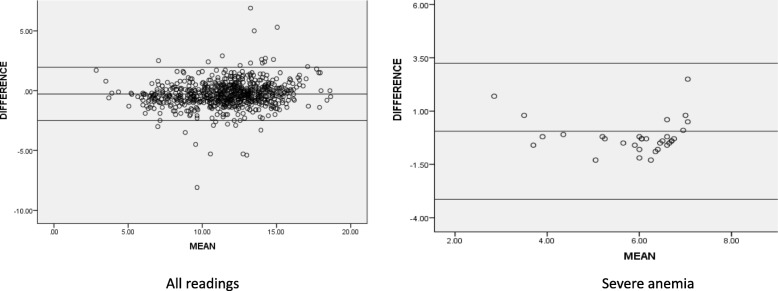
Bland-Altman Plot (Concordance analysis) showing agreement between auto-analyzer V/s venous sample for TrueHb (Hb values expressed as gm/dl)

The mean differences were calculated between autoanalyzer and index tests (using technician’s readings). It showed a bias or mean difference (95% CI) of 0.47 g/dl (0.42 to 0.52) for all readings, and 0.92 g/dl (0.82 to 1.03) for severe anemia with HemoCue. The sample showed a bias (95% CI) of − 0.28 g/dl (− 0.37 to − 0.20) for all readings, and 0.06 g/dl (− 0.52 to 0.63) for severe anemia with TrueHb. These results suggest that HemoCue overestimates, particularly in severe anemia, while TrueHb gives readings similar to the gold standard. (Table 2, Figs. 4, 5).

**Table 2:** Difference in Hb readings (mean, 95% CI) between autoanalyzer (reference) and index tests in different categories of anemia

Type of device	Device	All readings (Hb 2–20 g/dl)	Moderate and severe anemia (Hb < 10 g/dl)	Severe anemia (Hb < 7 g/dl)
**Invasive devices**	**Hemocue (venous)**	0.47 (0.42 to 0.52)	0.82 (0.71 to 0.92)	0.92 (0.81 to 1.02)
	**Hemocue (capillary)**	0.18 (0.11 to 0.25)	0.70 (0.58 to 0.82)	0.84 (0.66 to 1.03)
	**True Hb (venous)**	−0.28(− 0.36 to 0.20)	−0.23(− 0.41 to − 0.04)	0.05(− 0.52 to 0.63)
	**True Hb (capillary)**	−0.22(− 0.35 to − 0.09)	−0.23(− 0.43 to − 0.03)	−0.06(− 0.71 to 0.58)
**Non invasive devices**	**Massimo (technician)**	0.06(−0.10 to 0.24)	1.7 (1.55 to 1.97)	−0.39(−1.37 to 0.57)
	**Massimo (ANM)**	0.47 (0.37 to 0.58)	1.7 (1.56 to 1.98)	1.10 (0.20 to 1.99)
	**AJO (technician)**	0.43 (0.17 to 0.69)	2.6 (2.07 to 3.2)	3.84 (2.62 to 5.06)
	**AJO (ANM)**	0.71 (0.46 to 0.96)	2.5 (1.88 to 3.12)	4.1 (2.6 to 5.6)

### Reproducibility

Given that diagnostic accuracy of TrueHb and HemoCue devices were better compared to other devices, we assessed them for reproducibility by different raters of similar competence (two technicians), and agreement between users (ANMs v/s technicians). Both TrueHb and HemoCue both showed good reproducible results when different technicians tested the same sample using the same instrument. However, the level of agreement between technicians and ANMs showed better results with HemoCue (kappa 0.83; 95% CI 0.78–0.87 for all categories, 0.77; 95% CI 0.64–0.89 for severe anemia) as compared to TrueHb (kappa 0.62, 95% CI 0.56–0.68 for all categories, 0.73; 95% CI 0.63–0.82 for severe anemia).

### Performance of devices in different weather conditions (second phase)

Performance of TrueHb and HemoCue devices were assessed in different weather conditions (at four different sites- two sites from the first phase and two additional sites). The readings of TrueHb appeared to be more consistent although both HemoCue and TrueHb overestimated Hb by around 2 g% in extreme cold weather conditions. (Additional file 1: Webtable 3) This suggests that this factor should be borne in mind while using these devices in extreme cold weather conditions.

## Discussion

The evaluation of four point-of-care devices for measurement of Hb revealed that TrueHb and HemoCue performed better than Masimo’s device and AJO spectroscopic devices in the field settings. For detection of anemia, HemoCue and TrueHb were comparable while for severe anemia, TrueHb fared better than all other devices including HemoCue. However, both TrueHb and HemoCue overestimated Hb in extreme cold weather conditions.

Research staffs were trained in data collection and test procedures before commencement of data collection in a common session, and on site by the manufacturers, that increased the validity of results. They were blinded to the results of reference standard. Inter-observer variation or reproducibility for every device was assessed on a sample of 100 subjects. Also, to reduce intra-observer bias a group of three researchers collected the entire data at each site. The hospital laboratories where the study was conducted had mechanisms for external and internal quality assurance. The study population was drawn from pool of patients visiting the urban and rural health centres and general OPD attached to Medical Colleges. It was a diagnostic study with a pragmatic approach, considering technicians as well as health workers as end users of the devices.

However, the study suffers from certain limitations. We did not consider pregnant women and children in the study population due to ethical reasons. Also we excluded patients who had serious medical conditions many of whom might have had severe anemia. Although this was primarily done for ethical reasons, this could have introduced a selection bias. Given the constraints of time and resources, we restricted ourselves to few centres that might have affected the generalizability. Most of the data collection took place in warm and cold climatic conditions. We could not conduct the study in very hot and dry weather that limits the external validity. Besides, we used new devices for the study that makes us difficult to comment on the need for recalibration during their use.

Invasive devices have performed consistently better than non invasive devices [3, 17, 20]. Of all the devices available, HemoCue and Massimo’s devices have been evaluated in diverse settings and population groups. The accuracy of HemoCue has been found to be inconsistent [3, 10, 14, 15, 17, 21]. TrueHb has been evaluated in adult population in India in a single study with encouraging results [4]. Massimo have poor accuracy in most of the studies, although the precision was greater at higher levels of Hb, similar to our study [6, 13, 15–17, 22].

Differences exist between venous and capillary Hb estimation. For public health programs, measurement of Hb from capillary blood by community workers is operationally feasible and hence a more viable option. In our study, we found that agreement between venous and capillary Hb readings for True Hb was comparable while for HemoCue, capillary Hb performed better. This is in contrast to other studies where they found that the agreement between venous readings and autoanalyzers were better than capillary values [10, 14, 21]. While one school of thought is that capillary blood has an arterial source due to which Hb levels are higher than venous blood, others suggest that higher capillary Hb levels are due to hemoconcentration caused by the influence of posture [8]. A previous study comparing point-of–care devices highlighted that sensitivity of a test using capillary blood was better while specificity was better using venous sample [4]. Use of capillary blood is subject to errors depending on the skin thickness, depth and intensity of prick, temperature of skin and mixing up of serum. TrueHb method involves a controlled prick using a loading gun unlike HemoCue that probably was responsible for better accuracy. Besides, accuracy parameters may also vary depending on the underlying Hb distribution, according to a recent study [23].

Both TrueHb and HemoCue devices overestimated Hb by 1.5–2 g%. This is of concern since this can result in misclassification of categories that may affect management practices. HemoCue has been previously tested in hot weather conditions with good results [7]. Reports suggest that Massimo had low bias and high discrimination for the detection of excessive erythropoiesis in high altitude, and may be a useful point-of-care tool for large-scale surveillance in high-altitude settings [22].

Most of the prevailing methods for Hb estimation have been sufficiently validated in local settings. However, greater emphasis should be focused on their robustness in day-to-day operations, competency and skills required to obtain reliable outcomes, regular proficiency testing of the results, meticulous documentation and maintenance of data base [12]. In our assessment, Massimo and spectroscopic devices had better provisions and mechanisms to store data compared to TrueHb device. The current version of HemoCue used in the study did not have any provision to store data. Patient acceptability and ease of use was better with Massimo’s device compared to all other devices. Use of spectroscopic device entailed an objective measurement of readings but a lot of subjectivity ensued owing to the size of the equipment and skilful operation. For instance, the sensitivity of Massimo and Spectroscopic device for detection of anemia was 70.4 and 30.5% respectively in Kolkata while the same figures were 52 and 69% in Puducherry. Since human errors are likely to creep in during these processes, the technology needs to take into account the factors that affect measurement techniques. A device to qualify as the most effective one for community based programs should be valid, quick, easy to perform, user and patient friendly, portable, reagent free, pre-analytical error proof and operable by all. None of the devices tested fulfilled all the criteria. While TrueHb and HemoCue fulfilled most of them, yet both failed to give very accurate results in extreme cold weather conditions. Barring this fact, TrueHb seemed to be performing well in diverse locations and across the range of Hb. The non-invasive devices, though novel in their design, are not suitable replacement for invasive devices in their current forms. However, lessons learnt from the study will help to address the gaps and improve the future performance of every device. Besides, proficiency testing should be established for periodic monitoring of the results for quality, with internal quality controls and external quality assessment [12].

## Conclusions

Of the four point-of-care devices tested, TrueHb and HemoCue performed better than Massimo’s device and AJO spectroscopic device. For detection of anemia, True Hb and HemoCue were comparable. For severe anemia, True Hb seemed to be a better and feasible point-of-care device in the community settings.

## Supplementary information

**Additional file 1.**

## Data Availability

The dataset supporting the conclusions of this article are available with the corresponding author.

## Abbreviations

AIIMS: All India Institute of Medical Sciences
ANM: Auxillary Nurse Midwife
AUC: Area under the curve
CSPro: Census and Survey Processing System
Hb: Hemoglobin
ICMR: Indian Council of Medical Research
JIPMER: Jawaharlal Institute of Postgraduate Medical Education and Research
STARD: Standards for the Reporting of Diagnostic Accuracy Studies
