# Supplementary material for: Diagnostic accuracy of point-of-care devices for detection of anemia in community settings in India
Source: BMC Health Serv Res. 2020 May 26;20:468. doi: 10.1186/s12913-020-05329-9 (PMC7249358; doi:10.1186/s12913-020-05329-9)
Supplement: Supplementary file 1 — Additional file 1. [file 12913_2020_5329_MOESM1_ESM.docx]

**Webtable 1: Diagnostic Accuracy Parameters for testing anemia and no anemia as per ICMR classification**

| **Rater** | **Device** | **Sensitivity % (95% CI)** | **Specificity % (95% CI)** | **Positive predictive value (PPV)**  **(95% CI)** | **Negative Predictive value (NPV)**  **(95% CI)** | **Positive likelihood**  **ratio (LR+)**  **(95% CI)** | **Negative likelihood ratio (LR-) (95% CI)** | **Area under ROC**  **(95% CI)** |
| --- | --- | --- | --- | --- | --- | --- | --- | --- |
| **Technician** | **Hemocue**  **(n=622)** | 85.3^a,b,c^  (79.9-89.6) | 98.7^a,c^  (97.1-99.5) | 97  (93.5-98.9) | 93.1  (90.4-95.2) | 63.9  (28.8-141.8) | 0.15  (0.11-0.20) | 0.92^b,c^  (0.90-0.94) |
|  | **True Hb**  **(n=686)** | 92.8^d,e^  (88.7-95.7) | 88.1^d,e^  (84.9-90.9) | 79.1  (73.8-83.7) | 96.2  (94.0-97.8) | 7.82  (6.13-9.99) | 0.08  (0.05-0.13) | 0.90^d,e^  (0.88-0.93) |
|  | **Massimo (n=671)** | 62.5  ( 55.9-68.7) | 97.7^f^  (95.9-98.9) | 93.5  (88.5-96.9) | 83.1  (79.6-86.3) | 27.4  (14.7-51.0) | 0.38  (0.32-0.45) | 0.80^f^  (0.77-0.83) |
|  | **Spectroscopic Device**  **(n=575)** | 57.1  (49.6-64.3) | 76.5  (72.4-80.2) | 48.6  (41.8-55.5) | 82.0  (78.1-85.5`) | 2.43  (1.98-2.98) | 0.56  (0.47-0.67) | 0.67  (0.63-0.71) |
| **ANM** | **Hemocue**  **(n=632)** | 89.9%^a,b,c^  (85.1-93.6) | 93.3^a,b,c^  (90.5-95.4) | 86.7  (81.6-90.9) | 95  (92.5-96.8) | 13.37  (9.43-18.94) | 0.11  (0.07-0.16) | 0.92^a,b,c^  (0.89-0.94) |
|  | **True Hb**  **(n=663)** | 86.3^d,e^  (81.0-90.6) | 84.7^d,e^  (81.2-87.8) | 71.9  (66.0-77.2) | 93.2  (90.4-95.3) | 5.63  (4.54-6.99) | 0.16  (0.12-0.23) | 0.85^e^  (0.83-0.88) |
|  | **Massimo**  **(n=642)** | 66.0^f^  (59.2-72.4) | 97.5^f^  (95.5-98.7) | 92.6  (87.2-96.3) | 85.6  (82.2- 88.6) | 25.99  (14.39-46.96) | 0.35  (0.29-0.42) | 0.82^f^  (0.78-0.85) |
|  | **Spectroscopic Device**  **(n=575)** | 56.4  (48.8-63.7) | 75.4  (71.3-79.2) | 46.6  (39.8-53.4) | 82  (78-85.5) | 2.29  (1.87-2.81) | 0.58  (0.49-0.69) | 0.66  (0.62-0.70) |

a: Homocue vs True Hb (p<0.05); b: Homocue vs Massimo (p<0.05); c: Homocue vs Spectroscopy (p<0.05); d:True Hb vs Massimo (p<0.05); e: True Hb vs Spectroscopy (p<0.05); f: Massimo vs Spectroscopy (p<0.05)

**Webtable 2: Diagnostic Accuracy Parameters for testing severe anemia as per ICMR classification**

| **Type of Rater** | **Test** | **Sensitivity % (95% CI)** | **Specificity % (95% CI)** | **Positive predictive value (PPV)**  **(95% CI)** | **Negative Predictive value (NPV)**  **(95% CI)** | **Positive likelihood**  **ratio (LR+)**  **(95% CI)** | **Negative likelihood ratio (LR-) (95% CI)** | **Area under ROC**  **(95% CI)** |
| --- | --- | --- | --- | --- | --- | --- | --- | --- |
| **Technician** | **Hemocue**  **(n=622)** | 39.7^a,b,c^  (27.6-52.8) | 100^a,c^  (99.4-100) | 100  (86.3-100) | 94.1  (92.1-95.8) | - | 0.60  (0.49-0.74) | 0.70^a,b,c^  (0.64-0.76) |
|  | **True Hb**  **(n=686)** | 84.8^d,e^  (68.1-94.9) | 96.7^d^  (95.1-97.9) | 54.9  (40.3-68.9) | 99.3  (98.3-99.8) | 25.5  (16.6-39.1) | 0.16  (0.07-0.35) | 0.91^d,e^  (0.85-0.97) |
|  | **Massimo (n=671)** | 28.0  (16.2-42.5) | 99.7^f^  (98.8-100) | 87.5  (61.1-98.4) | 94.5  (92.5-96.1) | 86.9  (20.3-371.8) | 0.72  (0.61-0.86) | 0.64  (0.58-0.70) |
|  | **Spectroscopic Device**  **(n=575)** | 26.9  (11.6-47.8) | 96.3  (94.6-97.7) | 23.3  (9.9-42.3) | 97  (95.3-98.2) | 7.3  (3.48-15.61) | 0.76  (0.60-0.96) | 0.62  (0.53-0.70) |
| **ANM** | **Hemocue**  **(n=632)** | 46.8^a,b,c^  (34.0-59.9) | 99.2^a,c^  (98.1-99.7) | 85.3  (68.9-95) | 94.8  (92.7-96.4) | 56.3  (22.6-140.2) | 0.54  (0.42-0.68) | 0.73^a,b,c^  (0.67-0.79) |
|  | **True Hb**  **(n=663)** | 87.1^d,e^  (70.2-96.4) | 95.7^d^  (93.9-97.1) | 48.2  (34.7-62.0) | 99.4  (98.4-99.8) | 20.15  (13.77-29.5) | 0.13  (0.05-0.34) | 0.91^d,e^  (0.85-0.97) |
|  | **Massimo**  **(n=642)** | 17^f^  (7.6-30.8) | 99.8^f^  (99.1-100) | 88.9  (51.8-99.7) | 93.8  (91.7-95.6) | 101.2  (12.94-792.6) | 0.83  (0.73-0.95) | 0.58  (0.53-0.64) |
|  | **Spectroscopic Device**  **(n=575)** | 28  (12.1-49.4) | 95.9  (94-97.3) | 21.2  (9-38.9) | 97.1  (95.5-98.3) | 6.81  (3.27-14.16) | 0.75  (0.59-0.96) | 0.62  (0.53-0.71) |

a: Homocue vs True Hb (p<0.05); b: Homocue vs Massimo (p<0.05); c: Homocue vs Spectroscopy (p<0.05); d:True Hb vs Massimo (p<0.05); e: True Hb vs Spectroscopy (p<0.05); f: Massimo vs Spectroscopy (p<0.05)

**Webtable 3: Mean Hb difference across the sites for Invasive Devices *(device minus autoanalyzer)***

| **SITE** | **Hemocue** | | **TrueHb** | | **Months of data collection** | **Temp (^O^C),** Mean, median, range | **Humidity (%)**  Mean, median, range |
| --- | --- | --- | --- | --- | --- | --- | --- |
|  | **Venous**  Mean, SD, 95% CI | **Capillary**  Mean, SD, 95% CI | **Venous**  Mean, SD, 95% CI | **Capillary**  Mean, SD, 95% CI |  |  |  |
| **Kolkata** | **0.72**, 0.72; 0.64 to 0.79 | **0.43**, 1.17;  0.30 to 0.55 | **-0.07**, 1.06;  -0.19 to 0.04 | **-0.20**, 1.42;  -0.36 to -0.04 | July- Nov 2018 | 26;28;  19-29 | 77.4; 76;  69-87 |
| **Puducherry** | **0.23**, 0.60; 0.17 to 0.29 | **-0.09**, 0.97;  -0.19 to 0.01 | **-0.36**, 1.54;  -0.50 to -0.20 | **-0.15**, 2.14;  -0.35 to 0.05 | Sep 18- Jan 19 | 26.8;26.5;  25-29 | 81.2; 81;  77-86 |
| **Jodhpur** | **0.55**, 1.35; 0.35 to 0.75 | **-0.14**, 1.12;  -0.51 to 0.23 | **-0.14**, 0.84;  -0.25 to -0.02 | **-1.01**, 2.17;  -1.7 to -0.33 | Feb 19 | 28.3;28;  26-33 | 35; 35;  10-100 |
| **Kinnaur, Him Pradesh** | **2.16**, 0.49; 2.05 to 2.28 | **1.96**, 0.74;  1.79 to 2.17 | **1.62**, 0.88;  1.40 to1.84 | **1.83**, 1.35;  1.50 to 2.16 | Feb 19 | 0.2; 0.5;  -2 to 1 | 57 |
